# Hypermethylated PCDHGB7 as a Biomarker for Early Detection of Endometrial Cancer in Endometrial Brush Samples and Cervical Scrapings

**DOI:** 10.3389/fmolb.2021.774215

**Published:** 2022-01-04

**Authors:** Jiangjing Yuan, Zhanrui Mao, Qi Lu, Peng Xu, Chengyang Wang, Xiaona Xu, Zhaowei Zhou, Tongsheng Zhang, Wenqiang Yu, Shihua Dong, Yudong Wang, Weiwei Cheng

**Affiliations:** ^1^ Department of Obstetrics and Gynecology, International Peace Maternity and Child Health Hospital, Shanghai Key Laboratory of Embryo Original Diseases, and Institute of Birth Defects and Rare Diseases, Shanghai Jiao Tong University School of Medicine, Shanghai, China; ^2^ Shanghai Epiprobe Biotechnology Co., Ltd, Shanghai, China; ^3^ Department of Obstetrics and Gynecology, Jinshan Hospital of Fudan University, Shanghai, China; ^4^ Shanghai Public Health Clinical Center and Department of General Surgery, Huashan Hospital, Cancer Metastasis Institute and Laboratory of RNA Epigenetics, Institutes of Biomedical Sciences, Shanghai Medical College, Fudan University, Shanghai, China

**Keywords:** endometrial cancer, DNA methylation biomarker, *PCDHGB7*, cervical scrapings, endometrial brush

## Abstract

Endometrial cancer (EC) is one of the most common gynecologic cancers in developed countries. Presently, it is imperative to develop a reliable, noninvasive, or minimally invasive detection method for EC. We explored the possibility of using DNA methylation marker from endometrial brush samples (with a “Tao brush”) and cervical scrapes (with a “Pap brush”) for early detection of EC. We analyzed the methylation data of EC and normal endometrial tissues from The Cancer Genome Atlas (TCGA) and Gene Expression Omnibus (GEO) data sets. An optimized methylation-sensitive restriction enzyme combined with real-time fluorescent quantitative PCR (MSRE-qPCR) was used for methylation detection. Included in the training set were 143 endometrial tissues, 103 Tao, and 109 Pap brush samples. The validation set included 110 Tao and 112 Pap brush samples. *PCDHGB7* was significantly hypermethylated in EC compared with normal endometrial tissues in the TCGA and GEO data sets (AUC >0.95), which was verified in clinical samples. In the Pap brush samples, the AUC was 0.86 with 80.65% sensitivity and 82.81% specificity, whereas the Tao brush samples exhibited higher specificity (95.31%). The combination of Tao and Pap brush samples significantly increased the sensitivity to 90.32%. In the validation set, the final model yielded a sensitivity of 98.61%, specificity of 60.53%, positive predictive value of 82.56%, and negative predictive value of 95.83%. These results demonstrate the potential application of the novel methylation marker, hypermethylated *PCDHGB7*, in cervical scrapings and endometrial brush, which provides a viable, noninvasive, or minimally invasive method for early endometrial cancer detection across different clinical features and histologies to supplement current hysteroscopy diagnosis.

## Highlights

• This work reveals that *PCDHGB7* is hypermethylated in EC by bioinformatic analysis and clinical validation.
• We systematically evaluated the performance of hypermethylated *PCDHGB7* as a novel biomarker for EC detection in endometrial tissues and Tao and Pap brush samples.
• *PCDHGB7* hypermethylation functions as an early stage biomarker for its emergence at the early stage of EC progression.
• The robustness of hypermethylated *PCDHGB7* enables Tao and Pap brush samples to be minimally invasive or even noninvasive samples for early EC detection.

## Introduction

Endometrial cancer (EC) is one of the most common malignant tumors of the female reproductive tract worldwide (Sung et al., 2021). In view of the increasing prevalence of EC risk factors, such as diabetes and obesity in the general population, EC’s incidence is expected to increase to 82,000 and 122,000 new cases per year in 2020 and 2030, respectively (Rahib et al., 2014). EC is highly curable in the early stages with a 5-year overall survival of 95% for stage I disease, which is only 19% in stage IV. Although most EC occurs in postmenopausal women, there has been a recent significant increase of EC occurrence ranging from 2% to14% in women aged 40 years or younger (Duska et al., 2001; Ota et al., 2005; Nagase et al., 2019). For young women of childbearing age, if diagnosed in the early stage of EC without myometrial invasion and extrauterine spread, they still hold the opportunity to retain the uterus and/or ovaries. Therefore, early diagnosis of EC is crucial, which reduces female mortality and strives for treatment opportunities in younger patients to retain fertility or reproductive endocrine function.

Presently, effective early detection or screening methods for EC are urgently needed. The current clinical evaluation is not carried out until the patient has symptoms, such as abnormal uterine bleeding (AUB) or postmenopausal bleeding (PMB) (Clarke et al., 2018). Due to the lack of noninvasive or minimally invasive triage diagnosis, women with AUB or PMB have to undergo invasive procedures to obtain endometrial tissues by curettage and hysteroscopic biopsy to exclude EC. Most ECs appear to develop from endometrial hyperplasia (EH) to atypical hyperplasia (AH) with progress stretching for years. During the long course of disease progression, AUB and PMB patients endure invasive endometrial biopsies repeatedly. Only 5%–10% of them have an underlying EC or an EC precursor (Clarke et al., 2018), resulting in physical, psychological, and economic pressures.

The most common detection method for EC in postmenopausal women without clinical symptoms is transvaginal ultrasound (TVS), which measures endometrium thickness. However, it is not reliable to distinguish between benign and malignant endometrium due to its high false-positive rate (Jacobs et al., 2011). Considering serous ECs (SECs) with a poor prognosis can be present in the atrophic endometrium, which is often diagnosed at an advanced stage (Urick and Bell, 2019), EC can also occur in women without endometrial thickening. Therefore, it is necessary to develop a noninvasive or minimally invasive and effective molecular method for early detection of EC among symptomatic and asymptomatic high-risk populations, such as women with Lynch syndrome or increased BMI and tamoxifen users (Costas et al., 2019) to avoid a large number of unnecessary invasive diagnostic workups for most women with benign endometrium.

It is well-known that genomic and epigenomic alterations participate in carcinogenesis in various human organs (Baylin and Jones, 2016; Jones et al., 2016). DNA methylation changes are among essential epigenomic alterations leading to chromosomal instability and aberrant expression of tumor-related genes (Kanai, 2010; Ahuja et al., 2016). Accumulating evidence reveals DNA methylation as a promising cancer biomarker (Widschwendter et al., 2018). Previously, our group found that *PCDHGB7* was hypermethylated in various cancer types compared with their corresponding normal tissues, and hypermethylated *PCDHGB7* was identified as a universal cancer only marker (UCOM). (Dong et al., 2019; Dong et al., 2021). However, hypermethylated *PCDHGB7* has not been systematically investigated in EC until now. The shedding of EC cells and/or cell-free EC DNA into the lower genital tract provides an opportunity to leverage more sensitive molecular testing and less invasive biospecimen sampling methods comprising endometrial brushes (Wentzensen et al., 2014; Bakkum-Gamez et al., 2015), cervical scrapes (De Strooper et al., 2014; Huang et al., 2017; Liew et al., 2019), vaginal swabs, and vaginal tampons (Fiegl et al., 2004; Bakkum-Gamez et al., 2015; Sangtani et al., 2020).

Optimizing sample collection methods and identifying the molecular markers with the greatest sensitivity to detect EC and its precursors are all critical in the development of early detection among symptomatic and asymptomatic patients. In terms of sampling location, endometrial brush sampling is the closest to the neoplastic foci and can collect the most amount of DNA content in theory, and this has higher absolute methylation percentages compared with the cervical scrape samples also taken by physicians. However, the endometrial brush’s main flaw is that it is to be inserted into the uterine cavity with an unsuccessful insertion rate of 20% in nulliparous and 8% in parous women (Williams et al., 2008). Cervical scrape sampling is a relatively noninvasive and more convenient method, and it may be more accepted even for postmenopausal women.

The present study aimed to identify *PCDHGB7* methylation in EC and to further compare the performance of *PCDHGB7* hypermethylation in the detection of EC by cervical scrapes (with a “Pap brush”) with that of endometrial brush sampling (with a “Tao brush”) so as to provide evidence for adopting a more suitable, noninvasive or minimally invasive sampling method of DNA methylation markers in clinical settings for women with AUB or PMB and within the asymptomatic detection population.

## Materials and Methods

### The Cancer Genome Atlas and Gene Expression Omnibus DNA Methylation Data Analysis

The Illumina 450 K methylation array data from The Cancer Genome Atlas (TCGA) database was downloaded from the UCSC Xena browser (https://xenabrowser.net/). The absolute methylation values were calculated from the β values of a 450 K methylation array [methylation value = (β value +0.5) ×100%]. The only six probes (cg13933262, cg14011639, cg10435816, cg23563234, cg08938584, cg17011276) within the CpG island in the *PCDHGB7* promoter region were used, the final methylation value of each sample was calculated by the average of these six probes. The basic information and methylation levels of all samples from TCGA project are listed in Supplementary Table S1. The methylation array data sets (from the GSE136791, GSE93589, GSE67116, GSE33422, and GSE40032 series) from the Gene Expression Omnibus (GEO) database (https://www.ncbi.nlm.nih.gov/geo/) were downloaded. The absolute methylation values were calculated by the same method as above. A total of 11 probes (cg14011639, cg27487435, cg08938584, cg05558169, cg14378860, cg23563234, cg13933262, cg10435816, cg01643675, cg03510378, cg17011276) of EPIC methylation array, six probes (cg13933262, cg14011639, cg10435816, cg23563234, cg08938584, cg17011276) of 450 K methylation array, and two probes (cg14011639, cg23563234) of 27 K methylation array within the CpG island in the *PCDHGB7* promoter region were used, respectively, and the final methylation value of each sample was calculated by the average of all used probes. The basic information and methylation level of all samples from GEO database are listed in Supplementary Table S2.

### Clinical Samples Collection

A total of 577 samples, including 355 samples in the training set and 222 samples in the validation set (Supplementary Table S3), were collected from the International Peace Maternity and Child Health Hospital, School of Medicine, Shanghai Jiaotong University, Shanghai, China. The study was approved by the Ethics Committee of International Peace Maternity and Child Health Hospital. Informed consent was acquired from all patients and control subjects.

The benign endometrium (BE) group (normal endometrium (NE) and EH patients) was enrolled before hysteroscopy or hysterectomy, including women with an endometrium pathology diagnosis by hysteroscopy because of PMB, AUB, increased endometrial thickness, or by hysterectomy for benign indications. The EC and AH patients newly diagnosed by hysteroscopy or curettage and endometrial biopsy were prospectively enrolled prior to hysterectomy. Women were excluded if they had recurrent EC, other known cancers, or received neoadjuvant chemotherapy or radiotherapy. In the cases of EC and AH, patients underwent cervical scrape sampling with a Cervex-Brush^®^ (Rovers Medical Devices B.V., Oss, Netherlands) and endometrial brush sampling with a Tao brush (Cook Medical, Bloomington IN) by the physician before the day of hysterectomy or hysteroscopy, which were rinsed in SurePath^TM^ Preservative Fluid (TriPath Imaging^®^, Inc., Burlington, United States), respectively, and used for subsequent testing. Moreover, control group specimens were collected before their hysterectomy or hysteroscopy by the same method. Fresh-frozen tissue specimens were collected immediately after hysterectomy or hysteroscopy.

### DNA Extraction

Genomic DNA (gDNA) from endometrial tissue samples was extracted with the TIANamp Genomic DNA Kit (Tiangen Biotech, DP304), and gDNA from Pap brush and Tao brush samples was extracted with the EP Genomic DNA Kit (Epiprobe Biotech, K-21), both following the manufacturer’s protocol. The quantity and quality of gDNA were determined with Nanodrop 2000 (Thermo Scientific, Waltham, Massachusetts, United States).

### MSRE-qPCR and DNA Methylation Evaluation

Methylation-sensitive restriction enzyme-combined real-time fluorescent quantitative PCR (MSRE-qPCR) was used to detect *PCDHGB7* methylation as described previously (Dong et al., 2021). *GAPDH* gene-related region absence of any cutting sites was selected for normalization. For each digestion reaction, 100 ng gDNA was taken as input, and every two units of the HhaI (NEB, R0139) and HpaII (NEB, R0171) were added, making the final volume 25 μL, followed by digestion at 37°C for 30 min and heat inactivation at 95°C for 5 min. The subsequent dual real-time PCR was performed on the 7500 Real-Time PCR System (Life Technologies) with a program as follows: initiation at 95°C for 10 min, then 45 cycles of 94°C for 20 s and 60°C for 60 s. The DNA methylation level for each sample was evaluated by ΔCt = Ct__*PCDHGB7*_ – Ct__*GAPDH*_.

### Statistical Analysis

*p* values were calculated using the two-tailed nonparametric Mann–Whitney test by GraphPad Prism 7.0. ROC analysis was conducted using GraphPad Prism 7.0. The sensitivity, specificity, positive predictive values (PPVs), and negative predictive values (NPVs) were calculated by clinical calculator 1 (http://vassarstats.net/clin1.html).

## Results

### *PCDHGB7* Hypermethylation as a Biomarker in EC Detection

**FIGURE 1:**
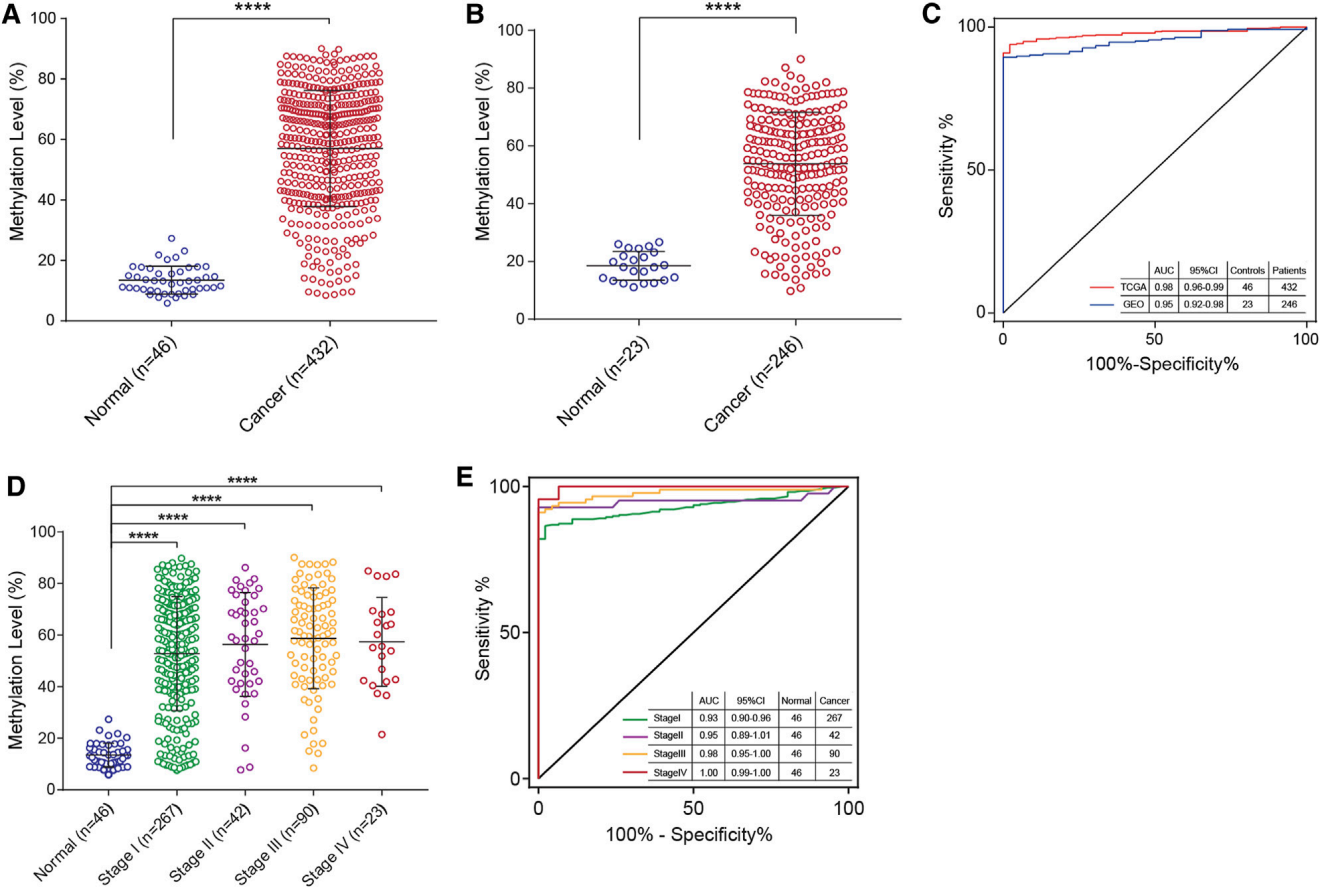
Hypermethylated *PCDHGB7* acted as a potential marker in EC in TCGA and GEO data sets. **(A)** *PCDHGB7* was hypermethylated in 432 EC endometrial tissues compared with 46 normal endometrial tissues in the TCGA database. **(B)** *PCDHGB7* was hypermethylated in 246 EC endometrial tissues compared with 23 normal endometrial tissues in GEO database. **(C)** The ROC curve of hypermethylated *PCDHGB7* in endometrial tissues from TCGA database and GEO database. **(D)** *PCDHGB7* methylation level in 468 endometrial tissues with determined stage from TCGA database. **(E)** The ROC curve of hypermethylated *PCDHGB7* in endometrial tissues with different stages from the TCGA database. In both **(A, B)** and **(D)**, *p* values were calculated using the two-tailed unpaired parametric test by GraphPad Prism 7.0. ****, *p* < .0001.

In our previous study, *PCDHGB7* was found to be hypermethylated in various types of cancer and identified as a universal cancer only marker (UCOM). Dong et al. (2019); Dong et al., 2021). Therefore, it is intriguing to investigate its potential application in alternative carcinoma detection sequentially. Methylation microarray data from the TCGA and GEO databases were analyzed. As expected, the methylation level of *PCDHGB7* in EC endometrial tissue samples was higher compared with normal tissue samples in 478 samples from the TCGA database and in 269 samples from the GEO database (Figures 1A,B). In addition, we evaluated the performance of *PCDHGB7* methylation as a biomarker for EC by receiver operating characteristic (ROC) analysis, and the results showed that the area under the curve (AUC) was 0.98 and 0.95, respectively (Figure 1C), suggesting that *PCDHGB7* hypermethylation holds potential to be a diagnostic marker for EC.

Moreover, to examine its probable application in early stage detection, we sorted 468 primary endometrial tissue samples with stages determined from the TCGA database and conducted the ROC curve analysis. The analysis shows significant differences of *PCDHGB7* methylation levels between normal and EC tissue samples of Stages I and II∼IV (Figure 1D). Meanwhile, the AUC of Stage I, II, III, and IV was 0.93, 0.95, 0.98, and 1.00, respectively (Figure 1E), suggesting that hypermethylation of *PCDHGB7* is a powerful marker for early stage ECs.

### Validation of *PCDHGB7* Hypermethylation in Endometrial Tissue Samples From EC Patients

Overall, 355 samples of the training set, including endometrial tissue samples, Pap brush samples, and Tao brush samples from 144 individuals, were included in this study, including 70 patients with NE, 29 patients with EH, 10 patients with AH, and 35 patients with EC, whereas 24 of 35 EC patients were in the early stage (Stage I). The statistics of samples refers to Supplementary Table S3. The age, BMI, histopathologic diagnosis, stage, and other clinics’ pathologic information for the participants are shown in Table 1. To assess whether the differences in age and BMI affect the methylation level of *PCDHGB7,* we analyzed the correlation between *PCDHGB7* methylation and age/BMI in both the TCGA database and clinical samples enrolled in the study. It turned out that the methylation level of *PCDHGB7* showed no relevance to age or BMI regardless of pathological groups (Supplementary Figure S1).

**TABLE 1:** Clinical characteristics of participants.

	Training set				Validation set	
	NE	EH	AH	EC	BE	EC
Number	70	29	10	35	39	73
Age, mean ± SD	51.87 ± 11.62	43.29 ± 9.24	50.01 ± 9.62	59.17 ± 9.97	52.24 ± 9.02	57.90 ± 9.80
BMI, mean ± SD	23.12 ± 3.39	21.80 ± 1.71	26.78 ± 5.23	24.22 ± 4.09	25.08 ± 3.10	25.41 ± 3.52
Hypertension and/or Diabetes, n (%)	19 (27.14%)	3 (10.34%)	4 (40.00%)	14 (40.00%)	11 (28.21%)	15 (20.55%)
FIGO 2009 Grade, n (%)						
Type I-G1	—	—	—	12 (34.29%)	—	29 (39.73%)
Type I-G2	—	—	—	11 (31.43%)	—	29 (39.73%)
Type II	—	—	—	12 (34.29%)	—	13 (17.81%)
Undetermined	—	—	—	0 (0.00%)	—	2 (2.74%)
FIGO Stage, n (%)						
IA	—	—	—	19 (54.29%)	—	41 (56.16%)
IB	—	—	—	5 (14.29%)	—	9 (12.33%)
II	—	—	—	0 (0.00%)	—	5 (6.85%)
IIIA	—	—	—	1 (2.86%)	—	0 (0.00%)
IIIC	—	—	—	6 (17.14%)	—	4 (5.48%)
IV	—	—	—	1 (2.86%)	—	0 (0.00%)
Undetermined	—	—	—	3 (8.57%)	—	14 (19.18%)
CA125, median (IQR)	15.70 (9.40, 21.40)	19.30 (11.35, 23.58)	17.15 (12.10, 20.48)	19.15 (11.46, 36.73)	15.95 (10.28, 23.18)	22.60 (16.30, 32.70)

NE, normal endometrium; EH, endometrial hyperplasia; AH, atypical hyperplasia; BE, benign endometrium; EC, endometrial cancer; IQR, interquartile range.

**FIGURE 2:**
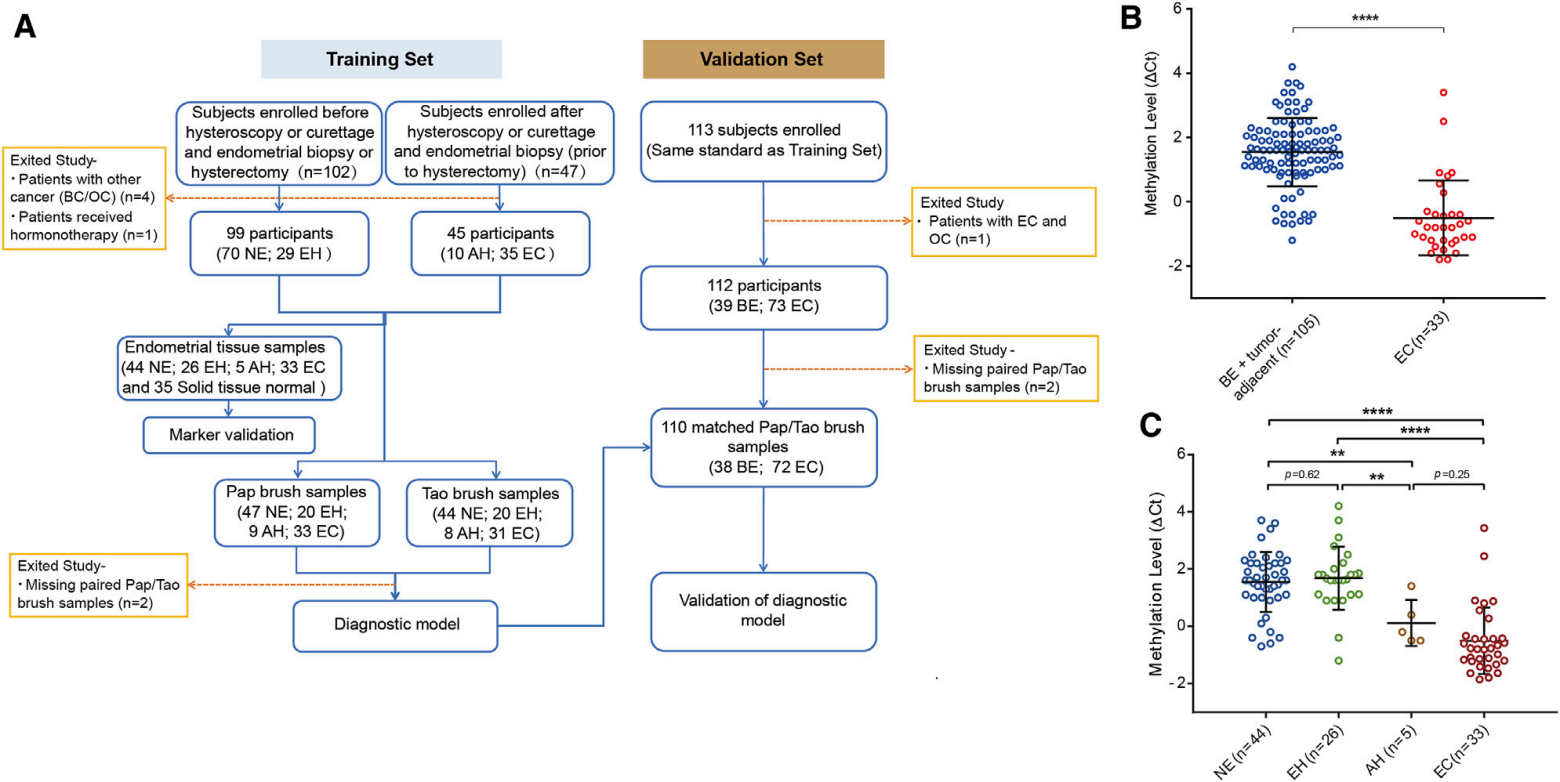
Methylation status of *PCDHGB7* had the capacity to make distinctions between BEs and ECs in clinical endometrial tissue samples. **(A)** The workflow of this study. NE, Normal endometrium; EH, Endometrial hyperplasia without atypia; BE, benign endometrium (NE + EH); AH, Atypical hyperplasia; EC, Endometrial cancer; BC, Breast cancer; OC, Ovarian cancer. **(B)** *PCDHGB7* methylation level in 138 endometrial tissue samples by MSRE-qPCR. **(C)** *PCDHGB7* methylation level in NE, EH, AH, and EC groups. In both **(B, C)**, *p* values were calculated using the two-tailed unpaired parametric test by GraphPad Prism 7.0. **, *p* < .01; ****, *p* < .0001.

The flowchart for the study is shown in Figure 2A. To evaluate the bioinformatics analysis results, we validated the methylation level of *PCDHGB7* in 33 endometrial cancer samples from EC patients, and 105 BE or tumor-adjacent endometrial tissue samples using MSRE-qPCR (Supplementary Table S4). As expected, the methylation level of *PCDHGB7* was found to be significantly higher in endometrial tissues from EC patients than in endometrial tissue samples of the BE group (Figure 2B). Additionally, we also found that *PCDHGB7* methylation was significantly higher in tissue samples from patients with EC/AH than patients with NE or EH (Figure 2C), demonstrating that detection of hypermethylated *PCDHGB7* using MSRE-qPCR contributes to early stage detection of EC by distinguishing AH from EH in endometrial tissue samples.

### Evaluation of *PCDHGB7* Hypermethylation in Pap Brush and Tao Brush Samples From Patients With EC and AH

**FIGURE 3:**
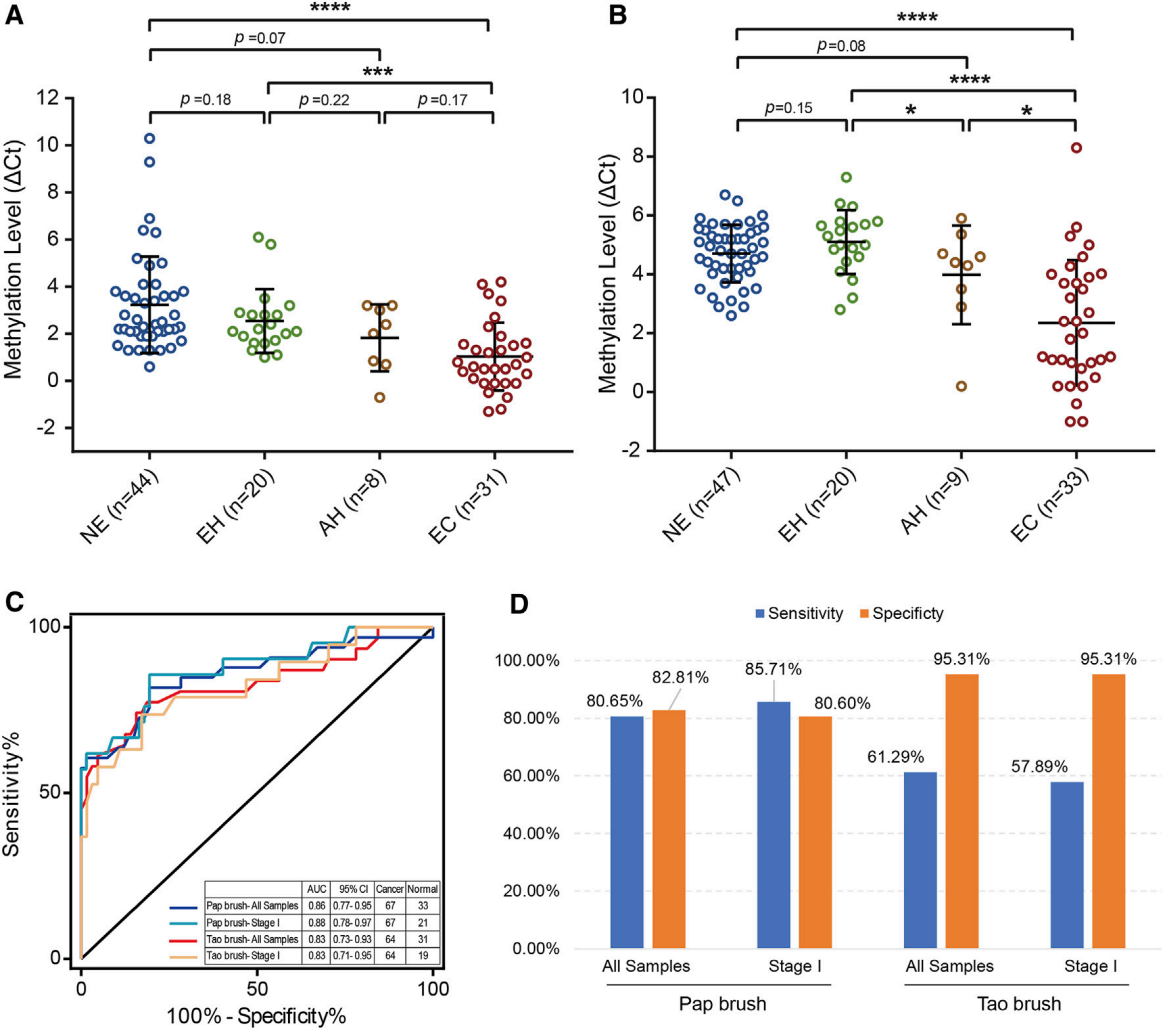
Detection of EC by using hypermethylated *PCDHGB7* in Pap brush and Tao brush. **(A)** *PCDHGB7* methylation level in 103 Tao brush samples by MSRE-qPCR. **(B)** *PCDHGB7* methylation level in 109 Pap brush samples by MSRE-qPCR. **(C)** The ROC curves of hypermethylated *PCDHGB7* in Pap brush and Tao brush samples for detecting ECs and early stage ECs. **(D)** The sensitivity and specificity of *PCDHGB7* hypermethylation in Pap brush and Tao brush samples. In both **(A, B)**, *p* values were calculated using the two-tailed unpaired parametric test by GraphPad Prism 7.0. *, *p* < .1; ***, *p* < .001; ****, *p* < .0001.

To make our EC detection method more applicable and cost-effective, we applied our assay in Tao brush samples of 44 patients with NE, 20 patients with EH, 8 patients with AH, and 31 patients with EC as well as Pap brush samples of 47 patients with NE, 20 patients with EH, 9 patients with AH, and 33 patients with EC (Supplementary Tables S3, S4). We found that *PCDHGB7* methylation was significantly higher in Tao brush samples from patients with EC than in NE and EH, whereas the difference between AH and EH showed no statistical significance (Figure 3A). However, significant differences between *PCDHGB7* methylation levels were found between Pap brush samples of AH and EH (Figure 3B). Furthermore, the clinical performance of *PCDHGB7* hypermethylation in the detection of EC from Pap brush and Tao brush samples were calculated. In Tao brush samples, *PCDHGB7* achieved ROC curves of 0.83 (95% CI = 0.73–0.93), and in Pap brush samples, it resulted in ROC curves of 0.86 (95% CI = 0.77–0.95) (Figure 3C). Meanwhile, the sensitivity and specificity of *PCDHGB7* methylation were 80.65% and 82.81% in Pap brush samples, whereas in Tao brush samples, the specificity (95.31%) was higher though with 61.29% sensitivity in the detection of EC (Figure 3D). Considering early stage EC is operable and largely curable, it is crucial to evaluate the robustness of hypermethylated *PCDHGB7* in early stage EC detection. As shown in Figure 3C, the performance of *PCDHGB7* hypermethylation in detection of Stage I EC (AUC = 0.88 in Pap brush and AUC = 0.83 in Tao brush) was comparable of it in detection of all EC samples, which was also supported by the sensitivity and specificity data in Figure 3D.

Moreover, similar to the TCGA database results, significant differences in *PCDHGB7* methylation levels can be found between EC and BE groups of early stage and early grade in Tao brush and Pap brush samples (Supplementary Figures S2A–S2D). Moreover, the methylation level of *PCDHGB7* was also significantly different in patients with tumor size less than 2 cm compared with BE groups (NEs and EHs) (Supplementary Figures S2E, S2F). Additionally, hypermethylation of *PCDHGB7* in Tao brush and Pap brush has also been observed in patients without myometrial invasion, vascular invasion, or AUB/PMB (Figure Supplementary Figures S2G–2L). These results support that detecting *PCDHGB7* hypermethylation in Tao brush and Pap brush samples are applicable in the early diagnosis of EC.

To further assess its application value in detecting EC, we compared the performance of hypermethylated *PCDHGB7* and the widely implemented EC biomarker, serum CA125 (Farias-Eisner et al., 2010). It demonstrated that serum CA125 of normal and cancer samples were largely overlapped (Supplementary Figure S3A). The inability of discriminating between normal and cancer samples indicates serum CA125 is a terrible marker for EC detection, which is further supported by the low AUC (0.61) in detecting EC (Supplementary Figure S3B). Under the cutoff (CA125 = 35 U/ml), the sensitivity of serum CA125 was 24.78%, which was far from satisfaction, though the specificity was 92.31%. The performance of hypermethylated *PCDHGB7* in both Pap and Tao brushes significantly outperformed serum CA125, suggesting that hypermethylated *PCDHGB7* was a robust marker for noninvasive or minimally invasive detection of EC.

### Construction and Validation of the Diagnostic Model for the Detection of Early Stage EC From Tao and Pap Brush Samples

We assessed whether the combination of hypermethylated *PCDHGB7* in Tao brush and Pap brush might provide better performance for EC early stage detection in terms of sensitivity and specificity. As shown in Table 2, the sensitivity and specificity (either positive as positive) of combined assay were 90.32% and 73.44%, respectively (PPV = 62.22%, NPV = 94.00%, accuracy = 78.95%). Furthermore, the sensitivity and specificity of both positive as positive were 61.29% and 100%, respectively (PPV = 100%, NPV = 84.21%, accuracy = 87.37%). Hypermethylated *PCDHGB7* was designated to be used for the early detection (screening or auxiliary diagnosis) of EC by using Pap or Tao brushes in the current study. In these application scenarios, sensitivity was the overarching priority; thus, the first model was chosen as the final diagnostic model.

**TABLE 2:** Performance of *PCDHGB7* methylation model in the detection of EC.

Diagnostic model	Endometrial cancer detection performance				
	Sensitivity (%)	Specificity (%)	PPV (%)	NPV (%)	Accuracy (%)
Pap Brush (Cutoff:<4.03)	80.65	82.81	86.49	62.69	84.21
Tao Brush (Cutoff:<1.25)	61.29	95.31	54.05	87.50	82.11
Either positive as positive (Cutoff: Pap<4.03, Tao<1.32)	90.32	73.44	62.22	94.00	78.95
Both positive as positive (Cutoff: Pap<2.5, Tao<4.55)	61.29	100.00	100.00	84.21	87.37

**FIGURE 4:**
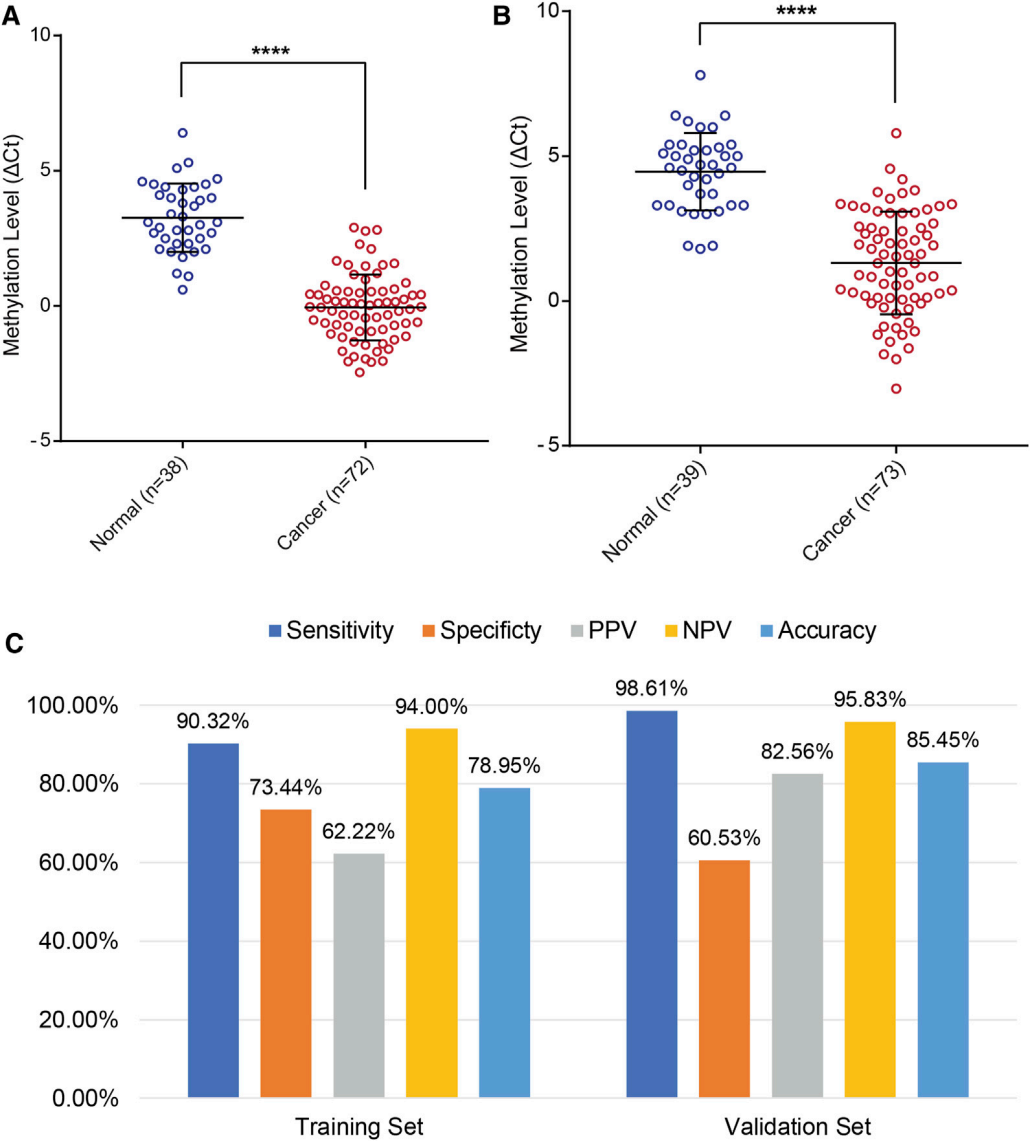
Validation of *PCDHGB7* performance for the detection of EC in validation set. **(A)** *PCDHGB7* methylation level in 68 Tao brush samples by MSRE-qPCR. **(B)**, *PCDHGB7* methylation level in 69 Pap brush samples by MSRE-qPCR. **(C)** The performance of *PCDHGB7* hypermethylation diagnostic model in training set and validation set. In both **(A, B)**, *p* values were calculated using the two-tailed unpaired parametric test by GraphPad Prism 7.0. ****, *p* < .0001.

In addition, the results of the training set were further validated in an independent testing set of 112 patients that included 73 EC and 39 BE groups (Supplementary Tables S3, S5). The EC group included 50 patients (68.49%) in the early stage (41 Stage IA, 9 Stage IB). The methylation level of *PCDHGB7* in Tao brush (Figure 4A) and Pap brush (Figure 4B) were both higher than those in controls. The clinical performance of *PCDHGB7* was calculated in the detection of EC from Tao brush and Pap brush, separately and collectively. The diagnostic model resulted in sensitivity of 98.61% and specificity of 60.53% (PPV = 82.56%, NPV = 95.83%, accuracy = 85.45%) (Figure 4C).

## Discussion

*PCDHGB7* is a member of protocadherins, which plays an inhibitory role in tumorigenesis and cancer progression by inducing cell cycle arrest and apoptosis (Hou et al., 2019). Previous studies demonstrate that *PCDHGB7* is crucial in the process of self-recognition and mutual recognition between synapses, the movement of synapses, and the establishment of the nervous system network (Sano et al., 1993; Frank and Kemler, 2002; Junghans et al., 2005; Morishita and Yagi, 2007). *PCDHGB7* is reported to be significantly hypermethylated in non-Hodgkin’s lymphoma (Shi et al., 2007). Besides, *PCDHGB7* hypermethylation gene was detected in approximately 80% of breast cancer, and *PCDHGB7* expression was reduced in breast cancer tissue (Shan et al., 2016a; Shan et al., 2016b). Importantly, we raised the concept of universal cancer only marker (UCOM) and identified hypermethylated *PCDHGB7* as a UCOM (Dong et al., 2019; Dong et al., 2021). This is the first study that enables *PCDHGB7* methylation in endometrial tissue and Pap/Tao brush samples for early EC detection. The great performance of UCOM, hypermethylated *PCDHGB7*, in noninvasive early detection of EC not only extends it clinical applications, but also proves the robustness of UCOM once again. The potential applications of UCOM in other cancer types deserve further investigation.

In this study, we verify that hypermethylated *PCDHGB7* can be used to early detect EC by Pap and Tao brush sampling even in EC patients of stage IA without myometrial invasion, G1, tumor < 2 cm in greatest dimension, lymphovascular space invasion (LVSI) (-) and without AUB/PMB (Additional file 2: Supplementary Figure S2). However, we did not observe different methylation levels of *PCDHGB7* in EC among various tumor sizes, histologic subtypes, and stages with or without myometrial invasion and LVSI, which reports similar results in previous studies (Bakkum-Gamez et al., 2015; Liew et al., 2019). It is indicated that ECs may share common epigenetic events, and that DNA methylation changes regardless of genetic heterogeneity and clinicopathology (Liew et al., 2019). However, it was encouraging that significant hypermethylated *PCDHGB7* could be detected via Pap or Tao brush in early low-risk EC patients of stage IA without myometrial invasion, G1, tumor < 2 cm in greatest dimension and LVSI (-), which are suitable for a fertility preservation procedure. Thus, DNA methylation marker *PCDHGB7* by noninvasive or minimally invasive sampling is promising for early diagnosis of EC in women of childbearing age, so these women can choose conservative alternatives to hysterectomy. Taken together, these data suggest that *PCDHGB7* hypermethylation may hold potential to increase the likelihood of women achieving disease-free survival and provide opportunities for the EC women of childbearing age to choose fertility preservation treatment. Moreover, monitoring of *PCDHGB7* methylation level from Pap/Tao brush samples may provide a convenient and noninvasive approach as a viable alternative to conventional endometrial surveillance during conservative treatment, which requires repeated endometrial biopsy every 3 months until the histological regression.

Pap brush and Tao brush samples are noninvasive or minimally invasive and conveniently obtained during routine office visits without anesthesia. The quality of the two samplings, which are taken by qualified physicians, is robust compared with cervicovaginal self-sampling methods. The Tao brush allows the sampler to be inserted to the intrauterine cavity, which is the closest to the anatomical sites of the tumors without injury to the myometrium or contamination from the endocervical canal (Liew et al., 2019). However, its main flaw is its unsuccessful insertion rate (Williams et al., 2008). In the present study, we failed to insert to the intrauterine cavity for seven women (2 EC, 1 AH, 4 BE). Therefore, we implemented cervical scrape sampling with Pap brush, which is aimed to sample the exocervical and endocervical canal before Tao brush sampling. Cervical scrapes are usually used for cervical cancer detection in the general population, causing minimal discomfort and hence being widely accepted by women. In our study, all cervical scrape specimens were taken successfully and yielded sufficient DNA for methylation analysis. Despite the results diverging from our expectations, we encouragingly found that the clinical performance of *PCDHGB7* hypermethylation in the detection of EC from Pap brush was more excellent than that from Tao brush samples evidenced by ROC curve analysis (AUC = 0.86 vs. AUC = 0.83), the sensitivity of *PCDHGB7* methylation in the Pap brush was superior to that of the Tao brush (80.65 vs. 61.29%), whereas the specificity of *PCDHGB7* methylation in the Tao brush was higher than that of the Pap brush as expected (95.31 vs. 82.81%). There are two possible explanations for this unexpected but enticing finding. One possible reason is that the sample size of our study is relatively small, and the marker performance could be influenced by clinical characteristics during population selection. The median tumor diameter was 2.6 cm (1.4, 4.1) in the present study, whereas the maximum tumor diameter < 2 cm was in 8 EC whose tumor may be localized to a small area of endometrium and may go undetected with the Tao brush. The other possible explanation is that the Tao brush has the disadvantage of not collecting enough endometrial cells of the uterine horns due to its round configuration (Du et al., 2016). The Pap brush was sampled from the cervix, serving as a conduit for malignant endometrial cells shedding from the whole uterine cavity, including uterine horns then gathering into endocervical canal, which could facilitate the sensitivity of Pap brush sampling method. Considering the individual advantages of Tao brush and Pap brush sampling, we suggest the combined diagnostic model (either positive as positive) if the Tao brush can be inserted into the intrauterine cavity successfully. Meanwhile, just using Pap brush specimens to detect *PCDHGB7* hypermethylation is acceptable as a complementary sampling method for those unsuccessful uterine cavity insertions as they are less invasive and more acceptable. In addition, we found significant *PCDHGB7* hypermethylation occurring within the Pap brush specimens of both AUB or PMB and asymptomatic EC women. As such, the utilization of the Pap brush for a DNA methylation marker as a biospecimen collection device carries promise as a means of detection in high-risk asymptomatic populations, particularly in postmenopausal women with atrophic uterus.

In spite of these novel findings, this study has a few limitations to be overcome. First, considering it was a single-center study, the inclusion of samples from multicenters will enhance the robustness of the conclusions. Moreover, the relatively small sample size of thevalidation set yielded high sensitivity and low specificity, suggested that the current diagnostic model might need a slight adjustment before direct application to clinical settings. In future studies, we propose to verify the clinical diagnostic accuracy in larger, prospective, and unbiased cohorts, especially for the AH group. Given endometrial surveillance, one should include repeated endometrial biopsy every 3 months until histological regression. Further studies are required to elucidate the role of the *PCDHGB7* methylation marker in surveillance of early EC or AH conservative treatment by a noninvasive or minimally invasive sampling method as well as consecutive surveillance of *PCDHGB7* methylation level using Pap or Tao brush sampling. Moreover, future multicenter clinical trials of *PCDHGB7* methylation via Pap and Tao brush sampling in women with AUB or postmenopausal bleeding and in high-risk asymptomatic populations should be planned.

## Conclusion

We demonstrate the usefulness of hypermethylated *PCDHGB7* as a biomarker for noninvasive or minimally invasive early EC detection. Combining the Tao brush with Pap brush sampling could acquire better detection performance, whereas a Pap brush would be more practical in detection settings in wider asymptomatic populations.

## Data Availability

All data generated or analyzed during this study are included in this article (and its Supplementary Material files).
