# Supplementary figures and images for: Hypermethylated PCDHGB7 as a Biomarker for Early Detection of Endometrial Cancer in Endometrial Brush Samples and Cervical Scrapings
Source: Front Mol Biosci. 2022 Jan 4;8:774215. doi: 10.3389/fmolb.2021.774215 (PMC8763697; doi:10.3389/fmolb.2021.774215)

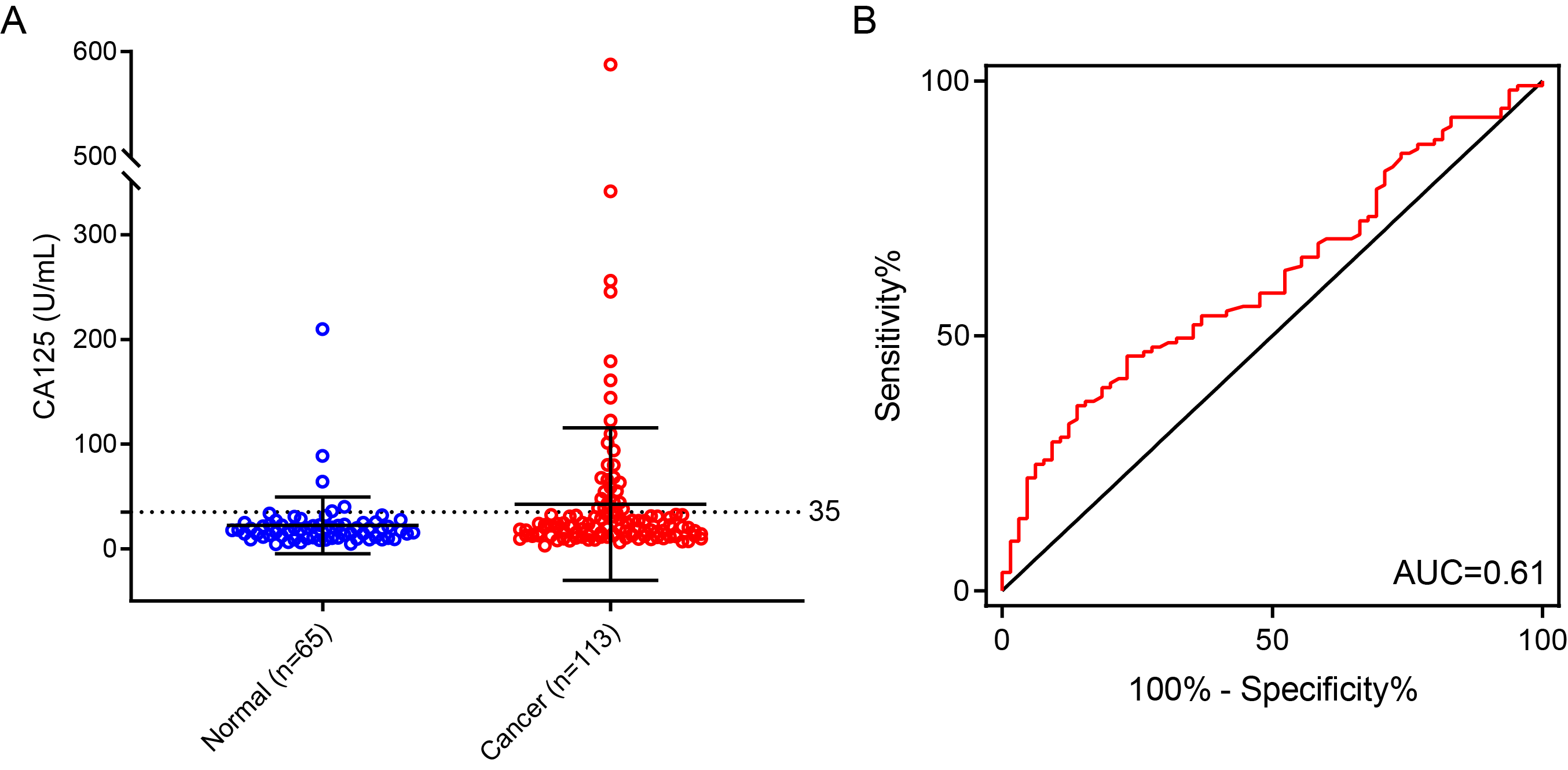

Supplement: Supplementary file 1 [file Image3.TIF]

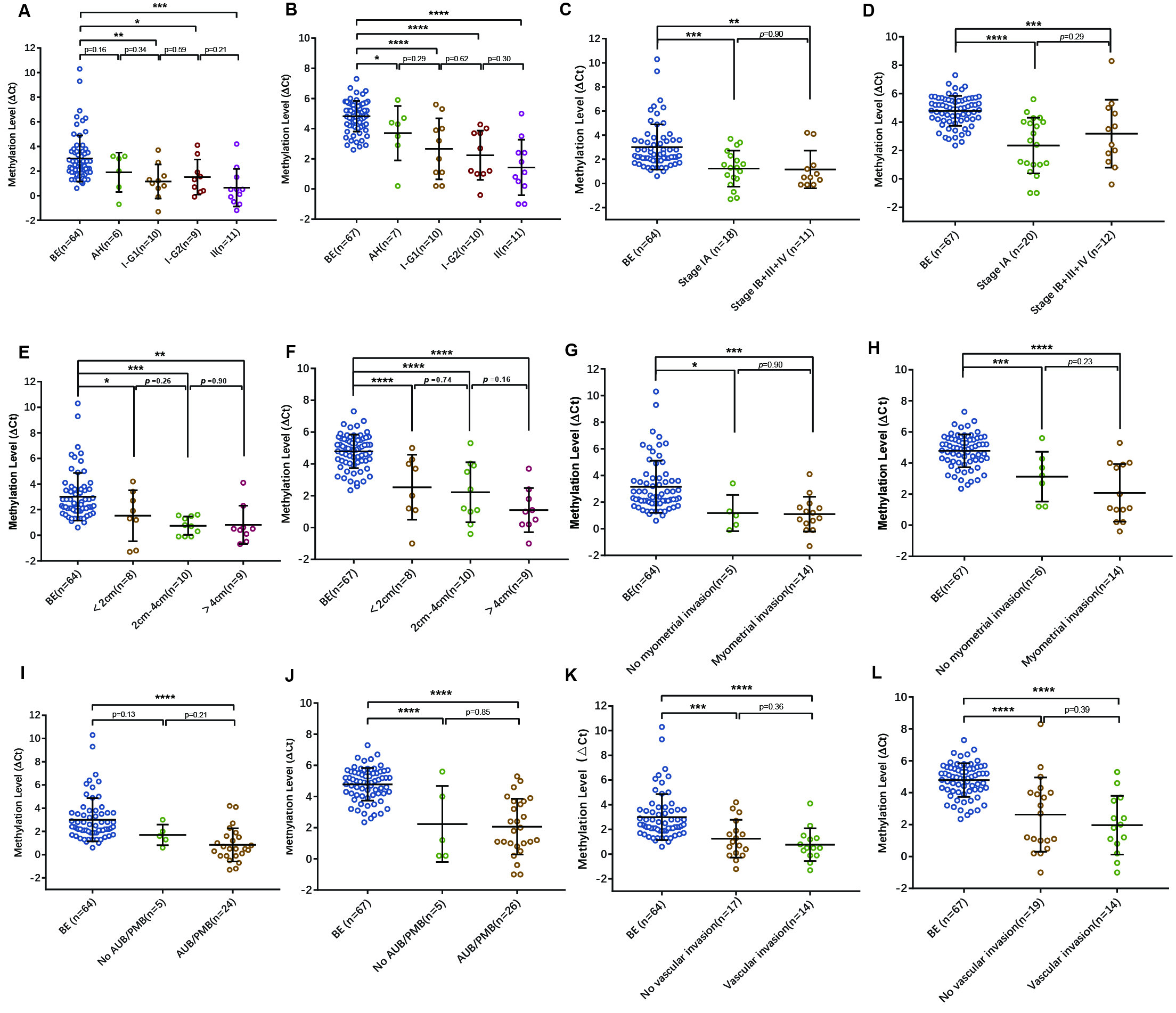

Supplement: Supplementary file 2 [file Image2.TIF]

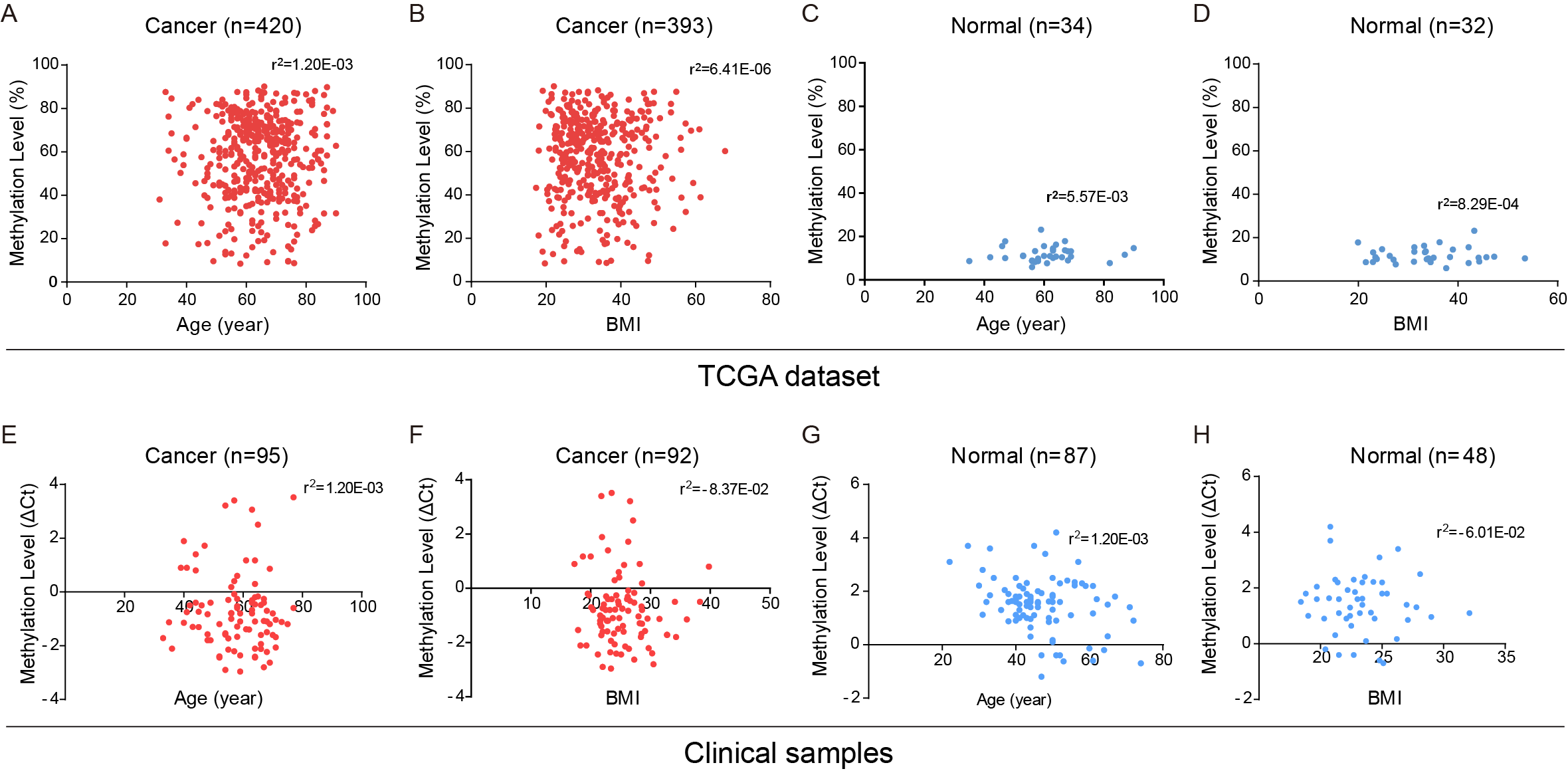

Supplement: Supplementary file 3 [file Image1.TIF]
